# Fas ligand and lytic granule differentially control cytotoxic dynamics of natural killer cell against cancer target

**DOI:** 10.18632/oncotarget.9980

**Published:** 2016-06-13

**Authors:** Yanting Zhu, Bo Huang, Jue Shi

**Affiliations:** ^1^ Center for Quantitative Systems Biology, Department of Physics and Department of Biology, Hong Kong Baptist University, Hong Kong, China; ^2^ School of Physics, Nanjing University, Nanjing, China

**Keywords:** immune-cancer cell interaction, natural killer cell, single cell dynamics, immunotherapy, cytotoxic lymphocyte

## Abstract

Interaction dynamics between Natural Killer (NK) cells and cancer targets have been the topic of many previous investigations, but the underlying rate-limiting kinetics and heterogeneity remain poorly understood. In this study, using quantitative single cell microscopy assay, we elucidate the differential dynamic control of NK-cancer cell interaction by multiple cytotoxic pathways. We found primary human NK cell, unlike NK cell line, killed adherent cancer target mainly by lytic granule-independent mechanism, in particular through Fas ligand (FasL). And the distinct kinetics of FasL and lytic granule pathway resulted in significant cell-to-cell variability. Killing by FasL occurred slowly, requiring transient, often multiple NK-cancer cell conjugations that gradually activated caspase-8, while lytic granule triggered rapid cytotoxicity by a switch-like induction of granzyme-B upon a single, prolonged conjugation. Moreover, interleukin 2 was observed to enhance both cytotoxic mechanisms by promoting target recognition by NK cell and increasing NK-cancer cell interaction frequency. Our results not only identify the key points of variation in the rate-limiting kinetics of NK-cancer cell cytotoxic interaction but also point to the importance of non-lytic granule mechanism for developing NK cell therapy.

## INTRODUCTION

Natural Killer (NK) cells are cytotoxic lymphocytes belonging to the innate immune system, capable of eliciting rapid immune response (usually within days) in the absence of antibodies and/or Major Histocompatibility Complex (MHC) molecules [1]. Upon target recognition by integrating signals from various inhibitory and activating receptors [2–6], NK cell unleashes its killing activities by both lytic granule-dependent and independent mechanisms [7]. Stored in the cytoplasm of NK cell, lytic granules contain membrane-disrupting protein, perforin, and proteases known as granzymes. These lytic granules can translocate into the target cell through the NK-target cell interaction juncture, after which granzymes are released to induce target cell death by proteolysis [8]. Death ligands, e.g., Fas ligand and TRAIL, on NK cell surface can also activate target killing, independent of lytic granule. Upon binding to death receptors on target cell, death ligands activate NK cell cytotoxicity by caspase-8 dependent, extrinsic apoptosis. Most of the previous NK cell studies have focused on understanding the interaction dynamics between NK and target cell through the lytic granule cytotoxic pathway [9–15]. Important questions remain to what extent as well as how the death ligand-mediated cytotoxicity, and possibly other non-lytic granule mechanisms, contribute to the kinetics and efficacy of NK cell killing.

Compared to cultured NK cell lines, such as NK92- MI and LAK [14–17], we found primary NK cells derived from human blood activated significantly less and much slower target cell death, even after pre-activation with Interleukin 2 (IL-2). Moreover, the killing activity of primary NK cells reduced further when against epithelial cancer target, as compared to the commonly used suspension target lines, e.g., K562 and 722.221 [12, 13]. Rate-limiting kinetics that govern the dynamic interaction between primary NK cell and adherent tumor target are clearly in need of further investigation. In this study, by quantitatively analyzing live-cell fluorescent reporters specific to distinct cytotoxic signaling pathways, we acquired novel single cell data that elucidated important questions regarding the kinetics and heterogeneity of NK- cancer cell interaction activated by multiple cytotoxic mechanisms simultaneously in action.

## RESULTS

### Cytotoxicity of primary NK cell is mainly independent of granzyme-B, in contrast to NK cell line

**Figure 1:**
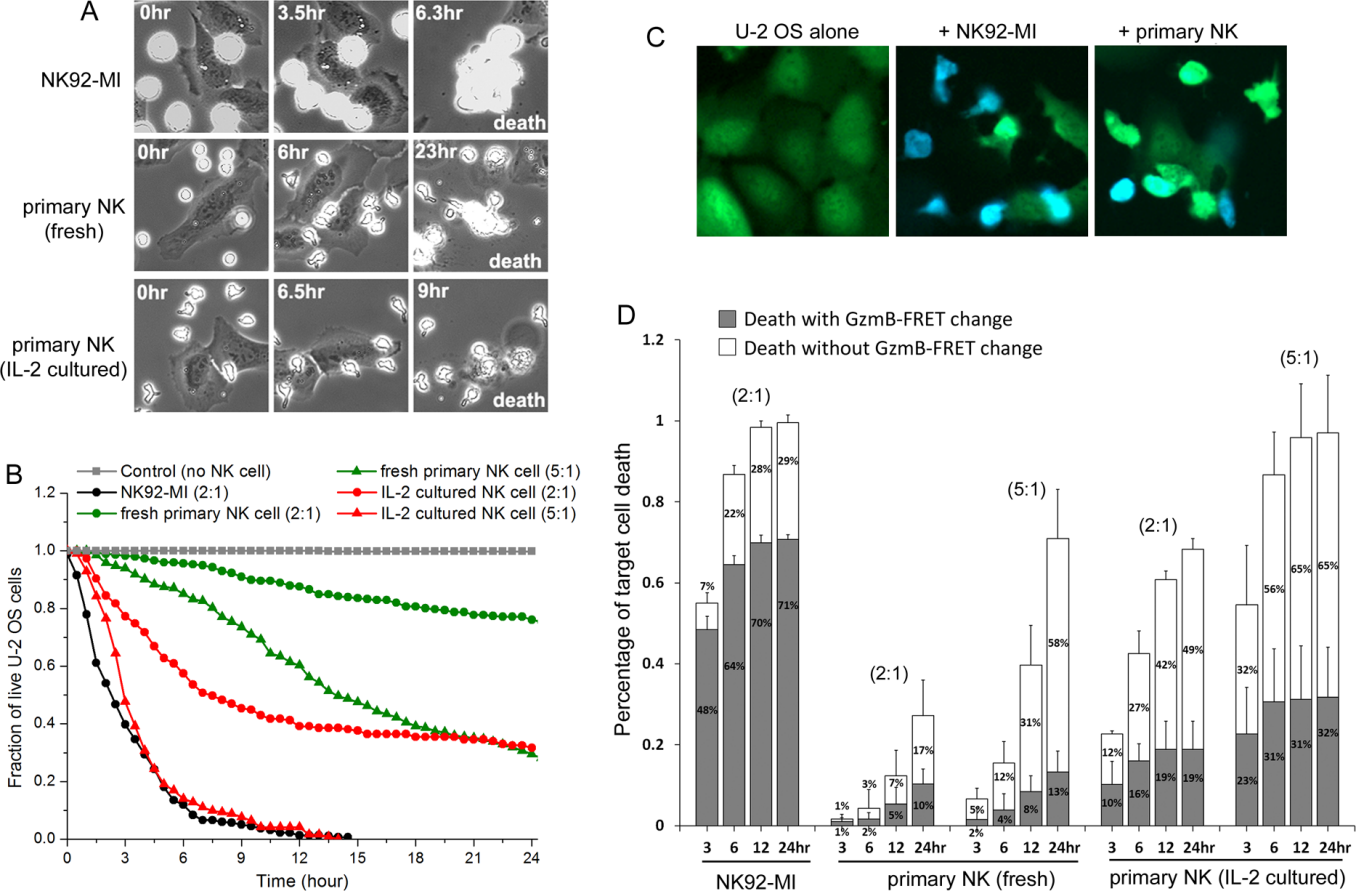
Cytotoxic dynamics of primary NK cells are distinct from NK cell line, NK92-MI (**A**) Phase-contrast images of distinct NK cells in co-culture with a human cancer cell line, U-2 OS, acquired from live-cell imaging. NK cells were added at time 0 (time is indicated in unit of hours at the upper corner of the still images). Cell death was scored by cell lysis. (**B**) Cumulative survival curves of target U-2 OS cells at the indicated co-culture condition: control (no NK cell, denoted in gray square); with NK92-MI at 2:1 NK-to-target ratio (black circle); with fresh primary NK cells at 2:1 (green circle) or 5:1 (green triangle) NK-to-target cell ratio; with 3-day IL-2 cultured primary NK cells at 2:1 (red circle) or 5:1 (red triangle) NK-to-target cell ratio. For all imaging experiments with primary NK cells, 50 ng/ml IL-2 was supplemented in the medium. Data were averaged from 3 independent imaging experiments and the number of cells analyzed ranges from 55 to 188, varied between conditions and experiments. Individual target U-2 OS cells were monitored by time-lapse microscopy, and the time from NK cell addition to morphological target cell death was analyzed and plotted as cumulative survival curves. (**C**) Fluorescent images of the granzyme-B FRET reporter from U-2 OS cells alone (1st column), U-2 OS in co-culture with NK92-MI for 10 hrs (2nd column) and U-2 OS in co-culture with 3-day IL-2 cultured primary NK cells for 10 hrs (3rd column). The images are overlay of the CFP (denoted by blue) and YFP (denoted by green) channels. (**D**) Distribution of the granzyme-B dependent (solid gray column) and independent (open column) killing of U-2 OS cells by different NK cells at the indicated co-culture conditions. The NK-to-target cell ratio is indicated at the top of the respective data set. Error bars: Standard deviations from 3 independent imaging experiments.

Given that the dynamic control of NK cells were mostly investigated using cultured NK cell lines, we first quantified cytotoxic dynamics of primary NK cell in comparison with a widely studied model NK cell line, NK92-MI, using live-cell imaging. Figure 1A shows time-lapse images of primary NK cells or NK92-MI in co-culture with a human epithelial cancer cell line, U-2 OS (derive from bone cancer) (Supplementary Movie SM1). The small, suspending NK cells are easily distinguishable morphologically from the large, adherent target cancer cell, U-2 OS. We scored target cell death by cell rounding and lysis and then plotted the killing kinetics as cumulative survival curves (Figure 1B). Three distinct dynamic features were immediately observable between primary NK cells and NK92-MI. Firstly, freshly isolated primary NK cells changed from ball-like shape to an elongated, polarized morphology after 6–10 hours in culture, while the majority of NK92-MI cells did not prominently exhibit such elongated morphology. Polarization is known to be important for NK cell activation, migration and target recognition [18]. The lack of polarization in NK92-MI did not appear to attenuate its killing efficiency, indicating that NK92-MI may be highly activated and thus do not require polarization, e.g., for target recognition. Secondly, contacts between primary NK and U-2 OS cells (scored by co-localization of the two cell types) were in general short (< 10 minutes), which was similar to previous observation [19]. However, NK92-MI often assumed very long contact (> 1 hour) with the target cells. Thirdly, at the same NK-to-target cell ratio (2:1), NK92-MI killed more than 20-fold faster than freshly isolated primary NK cells. Increasing the concentration of primary NK cells or pre-activating them with IL-2 for 3 days significantly increased their cytotoxic activity (Figure 1B).

Most literature on NK cell cytotoxicity focused on rapid target killing by way of lytic granule transfer and subsequent granzyme-B mediated proteolysis. The much slower killing kinetics that we observed with primary NK cells in culture led us to investigate whether the lytic granule/granzyme-B pathway played a lesser role in activating the cytotoxicity of primary NK cells as compared to NK92-MI. To monitor lytic granule/granzyme-B specific NK cell killing, we generated fluorescent U-2 OS reporter cell line that stably expresses a Förster Resonance Energy Transfer (FRET) construct, consisting of a cyan (CFP, donor) and yellow (YFP, receptor) fluorescent protein linked by a peptide substrate specific to granzyme-B, i.e., VGPDFGR [14]. Upon lytic granule transfer and release of granzyme-B into the target cell, granzyme-B cleaves the peptide linker of the FRET reporter, and energy transfer from CFP to YFP is thus lost, resulting in decrease of YFP fluorescence (denoted in green) and increase of CFP fluorescence (denoted in blue) (Figure 1C). Analysis of the FRET signal preceding target cell death showed that out of the 99% total cell death induced by NK92-MI after 24 hours of co-culture, about 71% were with a FRET signal change, indicating the cytotoxic process is mainly activated by the lytic granule pathway and granzyme-B (Figure 1D). However, out of the 71% total U-2 OS cell death induced by fresh primary NK cells (at 5:1 NK-to-target ratio), only 13% were preceded by the granzyme-B specific FRET signal change. Pre-activating primary NK cells with IL-2 for 3 days increased the overall target cell death to 97%, out of which about 32% were activated by granzyme-B, while the other 65% remained non-granzyme-B dependent. Similar dynamic characteristics were also observed with another target cancer cell line, HeLa (derived from cervical cancer), indicating that granzyme-B independent mechanism is responsible to activate major cytotoxicity of primary NK cell against at least some mammalian cancer targets (Supplementary Figure S1). Our results also point to important mechanistic difference in how primary NK cells elicits killing of adherent cancer target, as compared to the highly activated NK92-MI.

### Granzyme-B independent cytotoxicity of primary NK cell is mainly induced by death ligand, in particular the Fas ligand

**Figure 2:**
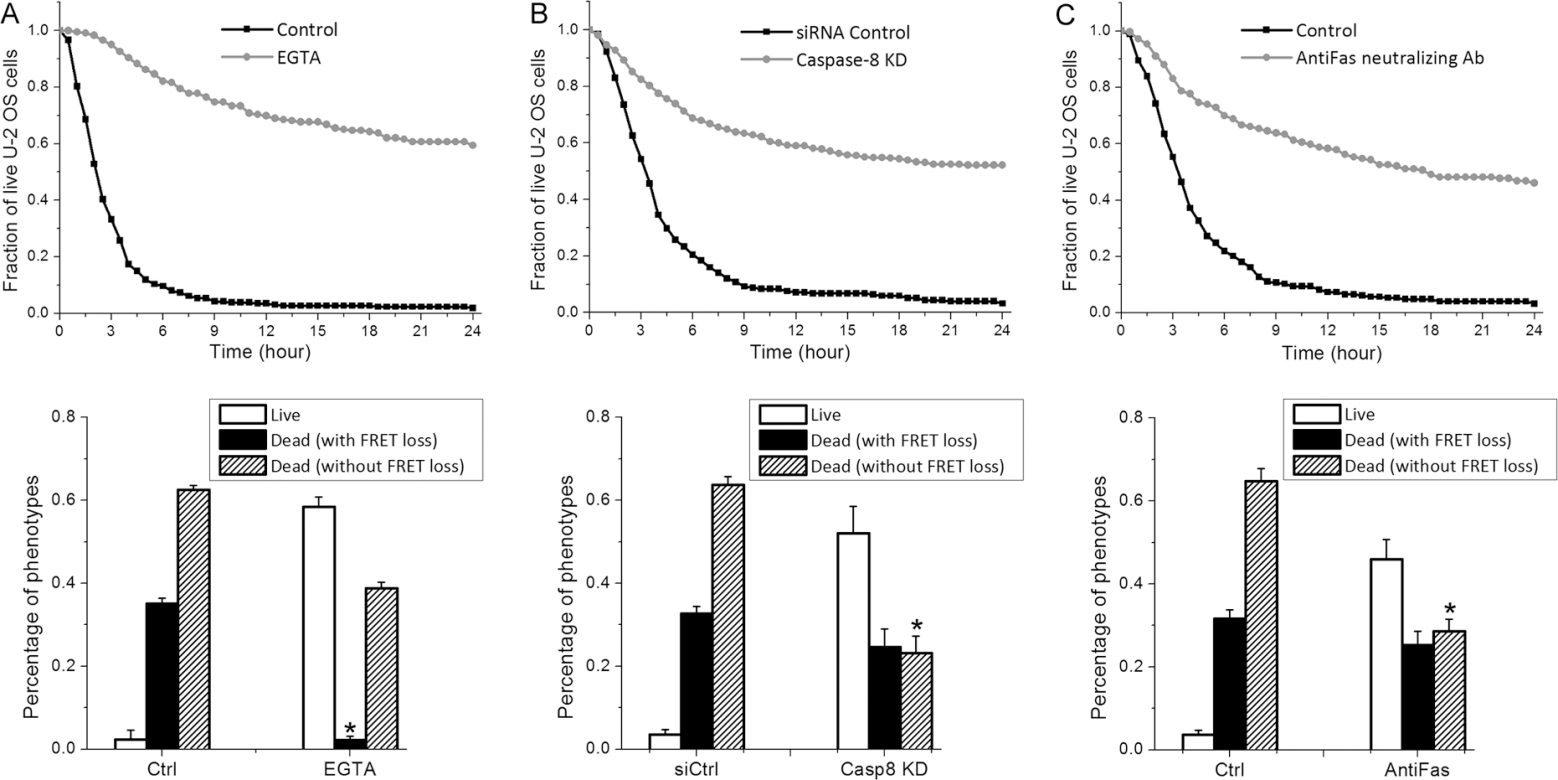
Granzyme-B independent killing by primary NK cells is mainly induced by extrinsic apoptosis through FasL Cumulative survival curves and distributions of granzyme-B and non-granzyme-B dependent target cell death quantified from U-2 OS cells in co-culture with primary NK cells (3-day cultured in IL-2) plus the indicated treatment: (**A**) 0.8 mM EGTA, (**B**) RNAi knockdown of caspase-8 and (**C**) 1.5 μg/ml anti-Fas neutralizing antibody. Data were averaged from 3 independent imaging experiments and the number of cells analyzed ranges from 64 to 155. Error bars: Standard deviations. *p < 0.001 vs. Control (Student's *t*-test).

The non-granzyme-B mediated cytotoxicity by primary NK cell could be induced by two possible mechanisms, one still involving lytic granule but different granzymes (e.g., granzyme M [20]), and the other involving extrinsic apoptosis triggered by death ligand. To examine whether lytic granule transfer is still responsible for target cell death not associated with granzyme-B activity, we used a calcium chelating regent, EGTA, to inhibit lytic granule transfer and measure the consequent NK cell activity. The presence of EGTA significantly attenuated the overall cytotoxicity of primary NK cells and abrogated nearly all target cell death activated by granzyme-B (Figure 2A), confirming the effect of EGTA in preventing lytic granule transfer and subsequent granzyme release into target cell. After 24 hours of NK- cancer cell co-culture in EGTA, about 40% of U-2 OS cell death that were independent of granzyme-B FRET signal change still occurred (Figure 2A), suggesting that the granzyme-B independent killing by primary NK cells is largely induced by cytotoxic pathway(s) not involving lytic granule transfer. Although the 40% granzyme-B-independent death under EGTA treatment is lower than the 60% observed under control condition after 24-hour co-culture, we note that the granzyme-B-independent death increased to about 54% at 36 hours and 63% at 48 hours of co-culture under EGTA, similar to the control condition (Supplementary Figure S2). EGTA treatment thus delayed the granzyme-B-independent death, but did not inhibit it. This result suggests that calcium flux may accelerate the granzyme-B independent death, although it is not required.

Activation of extrinsic apoptosis by death receptor binding is a second well-known, but less studied, mechanism by which NK cells elicit cytotoxicity. To determine whether extrinsic apoptosis is responsible for the granzyme-B independent target cell death, we knocked down caspase-8, the initiator caspase for extrinsic apoptosis, in U-2 OS cells by RNA interference (RNAi). Knockdown of caspase-8 significantly attenuated primary NK cell cytotoxicity, in particular reducing the percentage of target cell death that is independent of granzyme-B activity (i.e., no change in FRET signal) from about 63% to 23%, strongly suggesting that the majority of this population of cell death is activated by caspase-8 mediated extrinsic apoptosis (Figure 2B). As NK cells highly express Fas ligand (FasL) [21], we next investigated whether the observed extrinsic apoptosis is triggered by FasL binding to the death receptor, Fas/CD95, on U-2 OS cell surface. As shown in Figure 2C, silencing the FasL signaling pathway by an anti-Fas neutralizing antibody (1.5 μg/ml) particularly attenuated the granzyme-B independent NK cell killing, reducing the extent of U-2 OS cell death with no FRET signal change from about 65% to 28%, very similar to the degree of cytotoxicity attenuation observed with caspase-8 knockdown. In addition, blocking another well-known death ligand, TRAIL, by neutralizing antibody, did not attenuate granzyme-B independent target cell death (Supplementary Figure S3). Hence, our results illustrate that FasL is the major death ligand on primary NK cells that contributes to the activation of U-2 OS cell death through extrinsic apoptosis, although a remaining 23% of granzyme-B independent killing appeared to be activated by other non-lytic granule mechanism(s), possibly through cytokines, such as TNF-α and Interferon-γ [22, 23].

### Gradual caspase-8 induction triggered by transient FasL binding versus switch-like granzyme-B activation upon lytic granule transfer

The fact that primary NK cells simultaneously activates cancer cell death at least by two distinct cytotoxic pathways, i.e., FasL and lytic granule, raised intriguing questions regarding the rate-limiting kinetics of these two distinct killing modes and how their respective characteristics determine the likelihood of an individual target cancer cell die of one or the other cytotoxic mechanism. As indicated by results shown in Figure 1C, target cell killing by the granzyme-B dependent mechanism mostly occurred early, while a significant percentage of the non-granzyme-B dependent target cell death occurred at later time frames. Such kinetic difference between the two killing modes prompted us to further compare their induction characteristics in real time. The FRET signal specific to granzyme-B activation provides a convenient measure to quantify induction dynamics of the lytic granule pathway. In order to also monitor the activation dynamics of extrinsic apoptosis resulting from FasL binding, we engineered another U-2 OS reporter cell line that expresses a FRET construct, consisting of a CFP and a YFP linked by peptide substrate specific to caspase-8 [24]. In addition, we incorporated a red fluorescent reporter of mitochondria, IMS-RP [24], in both the caspase-8 and granzyme-B FRET reporter cell lines. As an abrupt transition from punctate to smooth localization of IMS-RP fluorescence corresponds to mitochondrial outer membrane permeabilization (MOMP), the committed step of apoptosis, the IMS-RP reporter allowed us to analyze the induction kinetics of primary NK cell killing (i.e., FRET signal change) relative to the precise onset of target cell death (i.e., MOMP) in real time. And we quantified the FRET signal as ratio of the CFP and YFP fluorescence in individual U-2 OS cell, i.e., an increase in FRET ratio corresponds to increase of the respective protease activity.

**Figure 3:**
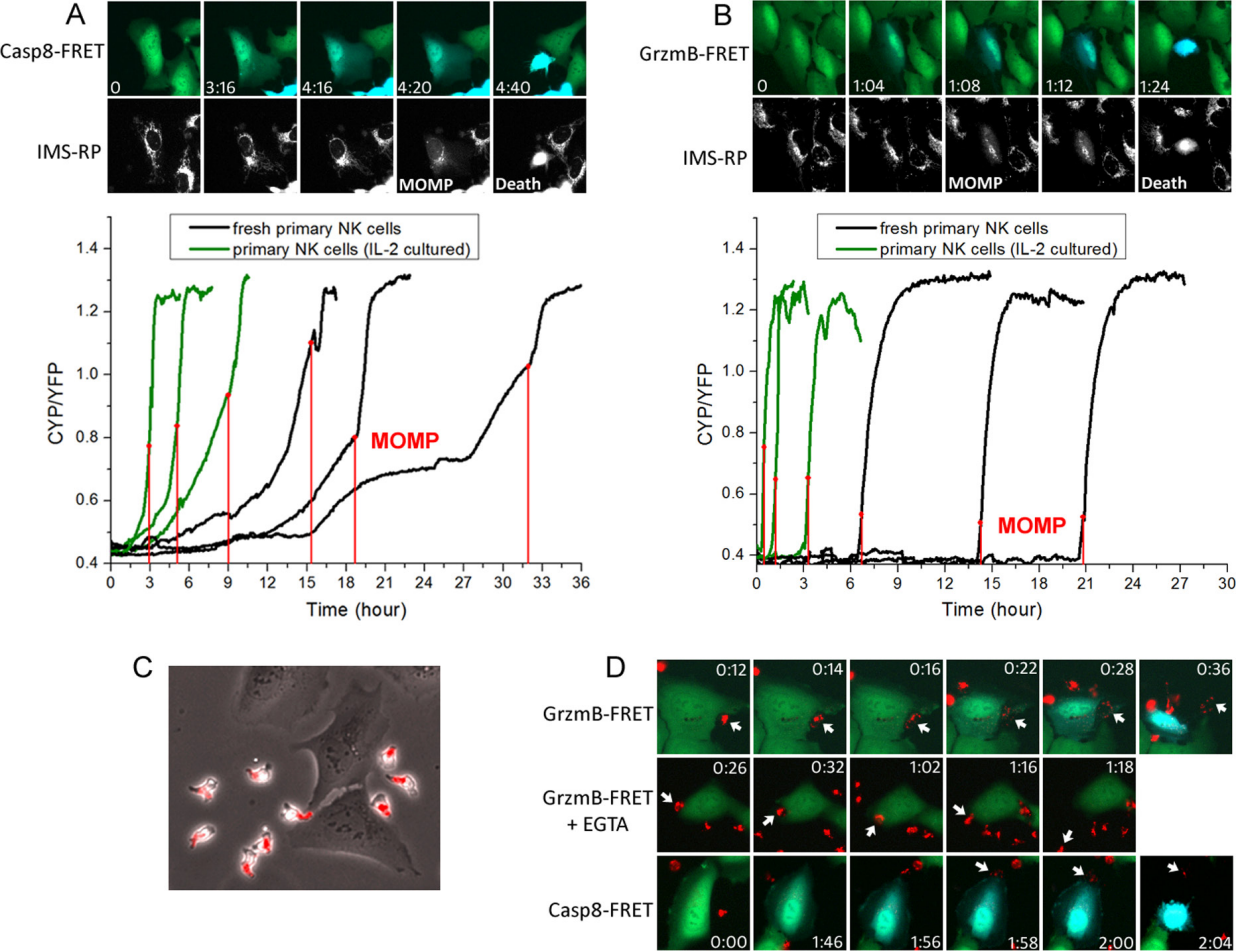
The FasL and lytic granule cytotoxic pathways exhibited distinct activation kinetics and involved different types of NK-target cell conjugation (**A**) Induction kinetics of caspase-8 by FasL. Upper panel: Still images were obtained from live-cell imaging of caspase-8 FRET reporter. The onset of apoptosis was scored by a change from punctate to smooth distribution in the fluorescence of the mitochondria reporter, IMS-RP (marked as MOMP on the corresponding image). Time is indicated in the unit of hour:minute. Lower panel: Representative time courses of the FRET signal ratio from individual U-2 OS cells, calculated as the ratio of CFP and YFP fluorescence from the caspase-8 FRET reporter. The time of MOMP is indicated by the red vertical line. (**B**) Induction kinetics of granzyme-B quantified based on fluorescence of the granzyme-B FRET reporter. (**C**) Localization of lytic granules in primary NK cells probed by an acidic granule marker, lysobrite. The images were overlay of the phase-contrast and red fluorescent channels. (**D**) Dynamics of lytic granule upon NK-target cell interaction under the indicated conditions. The images were overlay of the CFP (blue) and YFP (green) fluorescence from the respective FRET reporter and red fluorescence from lysobrite. The specific interacting NK cells are indicated by the white arrows.

The single cell trajectories revealed distinct activation dynamics of caspase-8 and granzyme-B preceding MOMP. The caspase-8 FRET ratio in individual U-2 OS cell exhibited a gradual increase before MOMP. There were multiple kinks in the single-cell trajectories of FRET ratio, especially from fresh primary NK cells, indicating that the rate of caspase-8 proteolysis increased in a step-wise manner. Such step-wise rate increase is most likely due to step-wise increase in the concentration of activated caspase-8, resulting from successful FasL-Fas conjugations with multiple NK cells in time (Figure 3A). Moreover, we found NK-U-2 OS cell conjugations preceding the increase of caspase-8 activity were always short, less than 4 minutes. Due to such transient nature of NK-U-2 OS conjugation that activates FasL signaling, very limited amount of caspase-8 is likely activated, thus requiring an extended period of accumulation, and also often multiple successful cytotoxic NK-U-2OS cell conjugations, to reach the threshold of MOMP. In contrast, the granzyme-B FRET ratio showed an abrupt, switch-like increase before MOMP. And this rapid induction of graznyme-B activity always involved a single, sustained NK-U-2 OS cell conjugation that typically persisted for 10–20 minutes (Figure 3B). Hence, primary NK cell killing by lytic granule transfer appeared to occur in an all-or-none manner. The long NK-target cell conjugation upon killing by lytic granule transfer is distinct from the transient conjugation for FasL-mediated cytotoxicity, which probably ensures extensive lytic granule transfer and activation of high level of granzyme-B activity to trigger rapid target cell death.

By staining the lytic granule with an acidic organelle marker, lysobrite, we next studied in more details the dynamics of lytic granule transfer relative to NK-target cell conjugation and the onset of protease activity. As shown in Figure 3C, the polarized primary NK cell stored lytic granules in its tail, as it moved and transiently interacted with the target U-2 OS cell. Such localization of lytic granules probably prevents undesirable leakage of lytic granules to the target in the absence of proper cytolytic conjugation during the constant transient contact between NK and target cells. Upon formation of a cytolytic conjugation, the lytic granules translocated to the leading front of NK cell, dispersed at the conjugation synapse, and then granzyme-B was released (indicated by FRET change), with each kinetic step taking about 2 minutes (Figure 3D; Supplementary Movie SM2). In the presence of EGTA, primary NK cells were still able to recognize and form sustained conjugation with the target U-2 OS cells. However, even though the lytic granules successfully translocated to the NK cell front, further dispersion and transfer into the target cells was abrogated due to the absence of calcium flux; and the NK cell eventually came off the target after 40 minutes to 1 hour (Figure 3D; Supplementary Movie SM3). Interestingly, by imaging the caspase-8 FRET reporter together with lysobrite, we found a number of U-2 OS cells exhibited a gradual induction of caspase-8 activity before encountering a sustained lytic NK-U-2 OS cell conjugation, followed by lytic granule transfer and rapid target cell death (Figure 3D; Supplementary Movie SM4). This observation revealed that a portion of U-2 OS cells that appeared to die of the lytic granule mechanism were in fact killed by the combined mechanism of FasL and lytic granule; and the FasL pathway contributes to lytic granule-mediated NK cell killing by activating caspase-8 and sensitizing the target cells to MOMP.

### Interleukin 2 promotes cancer target recognition by NK cell and increases NK-target cell interaction

**Figure 4:**
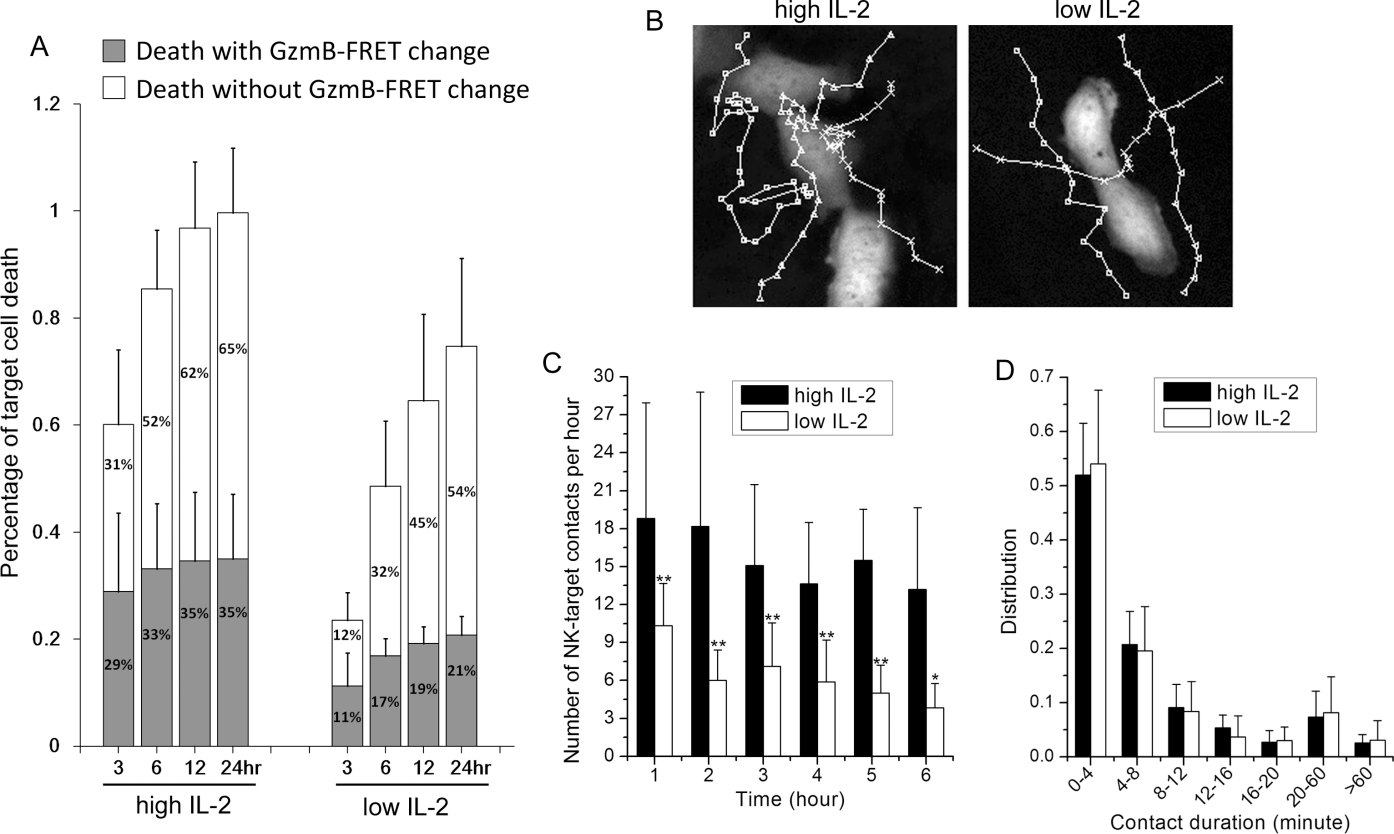
IL-2 promotes NK cell movement towards the cancer target and increases the frequency of NK-target cell interaction (**A**) Comparison of primary NK cell (3-day cultured in IL-2) cytotoxicity under high (50 ng/ml) and low (0.2 ng/ml) IL-2 supplement. Data were averaged from 4 independent experiments and the number of cells analyzed ranges from 51 to 84. Error bars: Standard deviations. (**B**) Representative NK cell trajectories, as they moved around the target cells under high vs. low IL-2. (**C**) Average number of contacts per hour (±SD) and (**D**) average contact duration (±SD) between a target U-2 OS cell and NK cells under high vs. low IL-2. Data for each condition were averaged from 15 individual target U-2 OS cells from 3 independent imaging experiments. *p < 0.03 vs. Control; **p < 0.005 vs. Control.

In all the experiments discussed above, we supplemented the primary NK-U-2OS cell co-culture with high Interleukin 2 (IL-2, 50 ng/ml), as IL-2 is a well-known cytokine that promotes NK cell survival and cytotoxicity. Previous studies have shown that the agonist effect of IL-2 on NK cell cytotoxicity is partly through activating expression of perforin, granzyme-B and FasL [25, 26]; and the transcriptional effect of IL-2 on NK cell motility and NK-target cell contact have been extensively characterized before [27]. Using the single cell imaging assay, we examined whether IL- 2, in addition to transcriptional activation of cytotoxic genes and receptors/ligands, activates other aspects of the dynamic control of NK-cancer cell interaction. We first compared the killing kinetics of primary NK cell (3- day cultured) under high (i.e., 50 ng/ml) versus low (i.e., 0.2 ng/ml) IL-2 supplement. Treatment condition of low IL- 2, instead of no IL-2, was chosen for the comparison, as we found primary NK cell did not survive well in culture in the absence of IL-2. High level of IL-2 induced more rapid and extensive U-2 OS cell killing (Figure 4A), and granzyme-B independent killing became an even more dominant cytotoxic mechanism under low IL-2 (Figure 4B). Given that the primary NK cells had been pre-activated by IL-2 for 3 days prior to experiments, the expression level of cytotoxic genes and surface receptors/ligands should be relatively similar. Therefore, the dose-dependent effect of IL-2 on NK cell cytotoxicity that we observed is likely due to additional, non-transcriptional regulatory mechanism(s).

One distinctive dynamic feature that we observed under low IL-2 is that primary NK cells generally assumed less contacts with the target cancer cells, as compared to that under high IL-2. Figure 4B shows representative NK cell trajectories near the target U-2 OS cell under high and low IL-2. NK cells under high IL-2 evidently stayed longer to the proximity of U-2 OS cells, indicating IL-2 promotes target recognition by NK cells. The average number of NK-U-2 OS cell contacts per hour (scored by co-localization) is plotted in Figure 4C. On average, 16 contacts per hour were observed between NK cells and a target U-2 OS cell under high IL-2, in comparison to 6 contacts per hour under low IL-2. In addition, contact frequency decreased in time more significantly under low IL-2. We observed no significant difference in the distribution of contact duration under high and low IL-2, with most NK-target cell interactions being of transient nature, persisting less than 4 minutes. In summary, our results suggest that in addition to transcriptionally activating cytotoxic genes and surface receptors/ligands, IL-2 also enhances NK cell cytotoxicity by promoting target detection by NK cells and increasing NK-target cell interaction frequency by non-transcriptional mechanism.

## DISCUSSION

The strong contribution that we observed from non-lytic granule cytotoxicity, e.g., activated by FasL, came as an unexpected result, as most of the available data reported on the dominant role of the lytic granule pathway. Our data showed that FasL signaling of NK cell not only directly activates cancer cell death but also sensitizes cancer cell to cytotoxicity induced by lytic granule. Moreover, cytotoxicity triggered by the FasL pathway outweighs the lytic granule mechanism even more, under low NK-to-target cell ratio (i.e., 2:1 as compared to 5:1) and low level of activating cytokine, IL-2, which is probably closer to the physiologically relevant condition. Although our findings have to be further examined and validated using more cancer types and animal model, they still point to a potentially critical role of the non-lytic granule pathway(s) and their associated recognition receptors in activating the cytotoxicity of primary human NK cell that need to be taken more into consideration, e.g., in the development of NK cell therapy. A recent study revealed an intricate control of tumor growth by NK cell uniquely through the FasL mechanism [28], suggesting that the FasL mechanism may indeed be exploited to provide new targets and strategies for engineering primary NK cells for adoptive cell transfer therapy.

Our data illustrated that not all transient NK-cancer cell interactions that were not immediately followed by target cell death were functionally futile, as some of them were successful FasL-Fas conjugations that led to caspase-8 activation. However, questions remain what kinetic and phenotypic determinants distinguish the FasL-Fas conjugations from most transient NK- cancer cell interactions that did not activate caspase-8. Mechanism of target cell recognition and formation of cytotoxic NK-target cell conjugation has been the subject of many previous studies, which revealed a complex signaling network involving various inhibitory and activating receptors on NK cell surface. We think these inhibitory and activating receptors are likely also involved to constrain or facilitate FasL-Fas conjugation, rendering variable outcome of the transient NK-target cell interactions. Further study to unravel the specific molecular regulators of FasL-Fas conjugation, e.g., by monitoring the FRET reporter together with fluorescent reporters of distinct surface receptors, is needed to improve our mechanistic understanding of the dynamic control by FasL signaling, and identify better cellular targets for engineering NK cells with enhanced killing efficacy.

We note the multiple cytotoxic mechanisms of primary NK cells are clarified by our findings, but not completely solved, as the combination of FasL and lytic granule-activated cytotoxicity did not account for 100% target cell death. About 23% U-2 OS cell death appeared to be independent of both lytic granule/granzyme-B and FasL/caspase-8 activity. One possibility is this minor population of target cell death is triggered by cytokine(s) secreted by NK cells upon recognition of the cancer target, such as TNF-α and Interferon-γ [21, 22]. Further study is needed to resolve the additional non-lytic granule cytotoxic mechanism(s). Nonetheless, whatever the precise mechanism is for the additional cytotoxicity, the important perspective from our study is that the predominant cytotoxic mechanism of primary human NK cells is mediated by death ligand, i.e., FasL, in addition to lytic granule; and that these two killing modes occur simultaneously via distinct kinetics, resulting in significant heterogeneity of NK cell activity.

## MATERIALS AND METHODS

### Primary human NK cell isolation and cell culture

Primary human NK cells were isolated from fresh peripheral blood (within 24 hours of blood donation) obtained from Hong Kong Red Cross. PBMCs were first prepared from the peripheral blood using Ficoll-Paque PLUS (GE Healthcare), from which NK cells were purified by negative selection using the EasySep Human NK Cell Enrichment Kit (Stemcell). Isolated human NK cells were either used immediately or cultured for 3 days at a density of 1 × 10^6^ cells/ml in RPMI 1640 medium containing 10 ng/ml recombinant human IL-2 (ThermoFisher), 10% heat-inactivated FCS, 100 U/ml penicillin and 100 μl streptomycin, prior to usage. The NK92-MI cell line was purchased from American Type Culture Collection (ATCC) and maintained in MyeloCult H5100 medium (Stemcell).

### Generation of fluorescent reporter target cell lines

The retroviral Förster Resonance Energy Transfer (FRET) construct that reports on granzyme-B proteolytic activity is a generous gift from Dr. Paul Choi (Genome Institute of Singapore) and consists of a cyan (CFP) and a yellow (YFP) fluorescent protein linked by a peptide substrate, VGPDFGR, specific to granzyme-B [14]. The FRET construct that reports on caspase-8 activity is a generous gift from Dr. Peter Sorger (Department of Systems Biology, Harvard Medical School) and consists of CFP and YFP fused by a peptide substrate, GLRSGGIETDGGIETDGGSGST, specific to caspase-8 [24]. U-2 OS or HeLa cells were either infected with the retroviral granzyme-B FRET construct or transfected with the regular caspase-8 FRET plasmid. Isogenic clones were then selected to generate the stable FRET reporter cell lines, U-2 OS-GrzmB (or HeLa-GrzmB) and U-2 OS-Casp8 (or HeLa-Casp8). We further infected the reporter cell lines with a retroviral construct, IMS-RP, which encodes a monomeric red fluorescent protein targeted to the inter-membrane space of mitochondria by fusion to the leader peptide of SMAC (a generous gift again from Dr. Peter Sorger [24]). U-2 OS and HeLa reporter cells were routinely maintained in McCoy's 5A medium and DMEM medium, respectively, with supplement of 10% heat-inactivated FCS, 100 U/ml penicillin and 100 μl streptomycin.

### Chemicals, neutralizing antibody and siRNA oligo

EGTA from Sigma was used at 0.8 mM. Lysobrite for staining the lytic granule was from AAT Bioquest (Sunnyvale, CA, USA). The anti-Fas neutralizing antibody (human, clone ZB4, EMD Millipore) was used at 1.5 μg/ml. Recombinant human TRAIL (#752902, Biolegend) and anti-TRAIL neutralizing antibody (#308202, Biolegend) were used at 0.5 μg/ml and 1.5 μg/ml, respectively. siRNA oligo to silence caspase-8 (5′-UGGAUUUGCUGAUUACCUAuu-3′) was custom synthesized by Dharmacon and used at final concentration of 40 nM. Dharmacon On-Target plus siControl (D- 001810- 01) was used as non-targeting siRNA control. All siRNA transfections were performed using HiPerFect (Qiagen). Experiments were conducted after 48 hrs of gene silencing.

### Time-lapse microscopy

Target U-2 OS or HeLa cells were plated in 24- well glass-bottom imaging dish (MatTek, USA) one day before the imaging experiments. On the day of imaging experiment, primary NK cells were added into the imaging dish at defined NK-to-Target ratio and the two cell types were co-cultured in phenol red-free CO_2_-independent medium (Invitrogen) supplemented with 10% heat-inactivated FCS, 100 U/ml penicillin, 100 μl streptomycin and 50 ng/ml or 0.2 ng/ml IL-2. Cell images were acquired using the Nikon TE2000-PFS inverted microscope enclosed in a humidified chamber maintained at 37°C. Cells were imaged every 2, 4 or 10 minutes (varied between experiments), using a motorized stage and a 20X objective (NA = 0.95). To quantify the cumulative survival curves and the phenotype distributions, we viewed and analyzed the images manually, using the MetaMorph software (Molecular Dynamics). Based on morphology, we scored cell death by blebbing followed by cell lysis. The cytotoxic mode was scored based on granzyme-B or caspase-8 specific FRET signal. To quantify the time courses of FRET signal, we used an automatic cell tracking program that we developed using Matlab. The program consists of image analysis procedures that sequentially segment the individual cells, track them in time, as well as measure and ratio the cellular CFP and YFP fluorescence intensity.

## SUPPLEMENTARY MATERIALS










