# Supplementary material for: Fas ligand and lytic granule differentially control cytotoxic dynamics of natural killer cell against cancer target
Source: Oncotarget. 2016 Jun 13;7(30):47163–72. doi: 10.18632/oncotarget.9980 (PMC5216932; doi:10.18632/oncotarget.9980)
Supplement: Supplementary file 1 [file oncotarget-07-47163-s001.pdf]

Fas ligand and lytic granule differentially control cytotoxic dynamics of natural killer cell against cancer target

Supplementary Materials

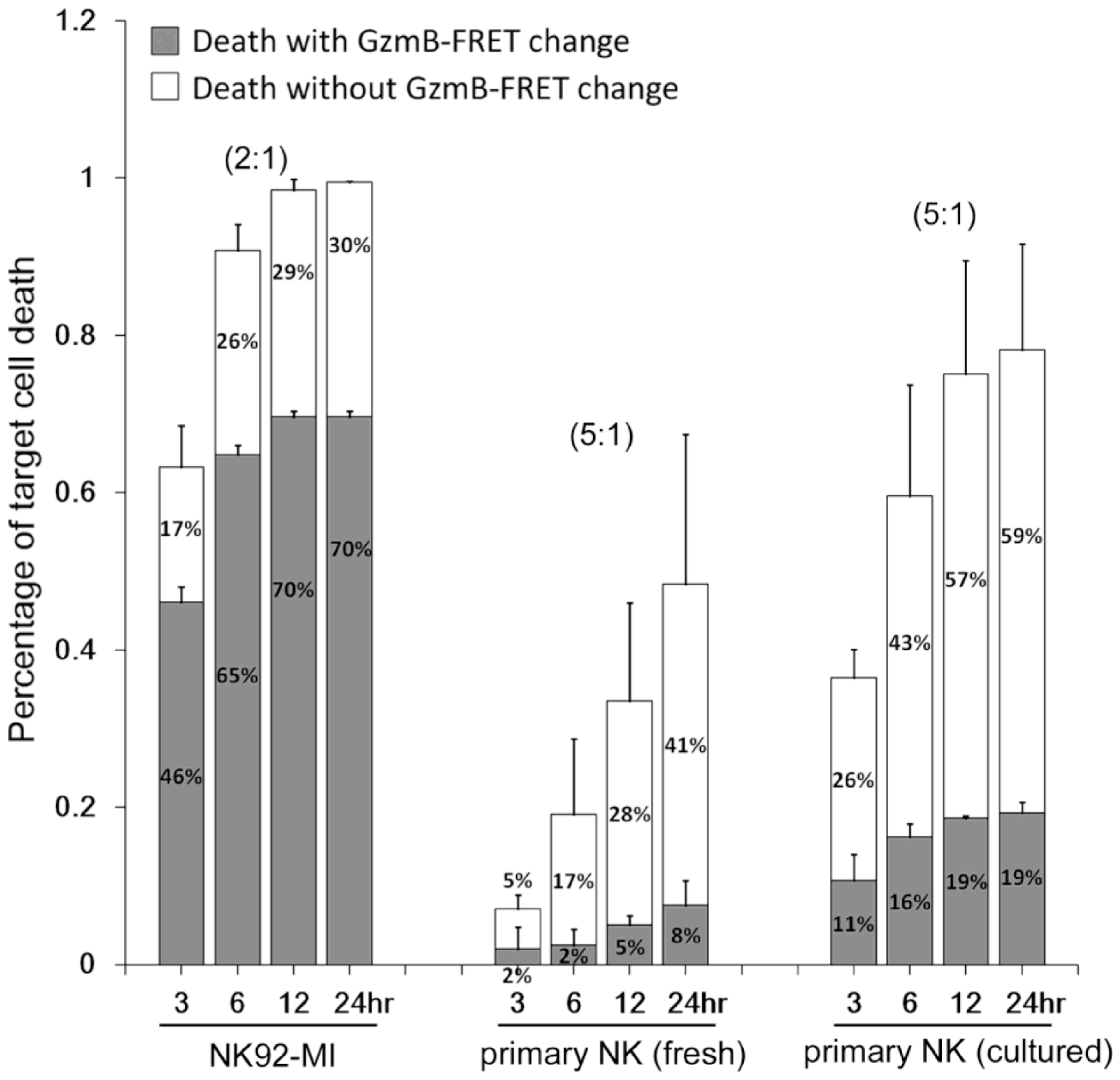

Supplementary Figure S1: Distribution of the granzyme-B dependent (solid gray column) and independent (open column) killing of another adherent cancer cell line, HeLa, by primary NK cells or NK92-MI at the indicated NK-to-target cell ratio. Error bars: Standard deviations of two independent imaging experiments. The number of cells analyzed ranges from 51 to 97, varied between conditions and experiments. The data demonstrated the dominant role of granzyme-B independent killing of HeLa cells by primary NK cells, which is similar to results acquired for U-2 OS cells.

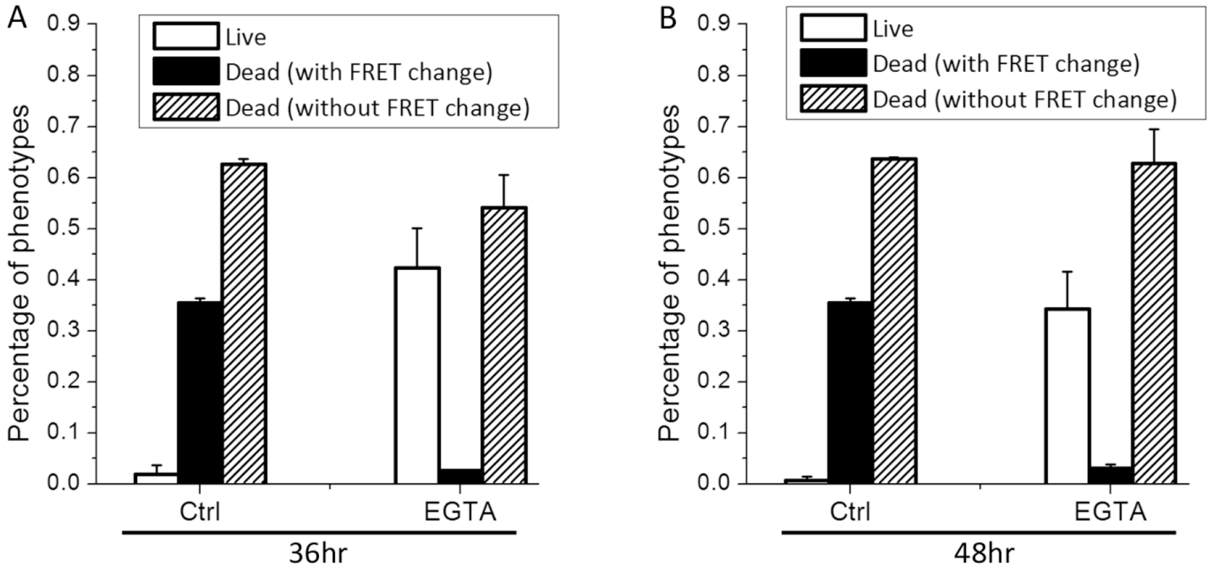

Supplementary Figure S2: Distributions of granzyme-B and non-granzyme-B dependent target cell death quantified from U-2 OS cells in co-culture with primary NK cells (3-day cultured in IL-2) plus 0.8 mM EGTA for (A) 36 hours or (B) 48 hours. Data were averaged from 3 independent imaging experiments and the number of cells analyzed ranges from 72 to 88. Error bars: Standard deviations. The data showed that the percentage of granzyme-B independent cell death under EGTA treatment increased to a level similar to that under the control condition after 36–48 hours of NK-target cell co-culture. Therefore, EGTA treatment delayed, but did not inhibit, granzyme-B independent NK cell killing.

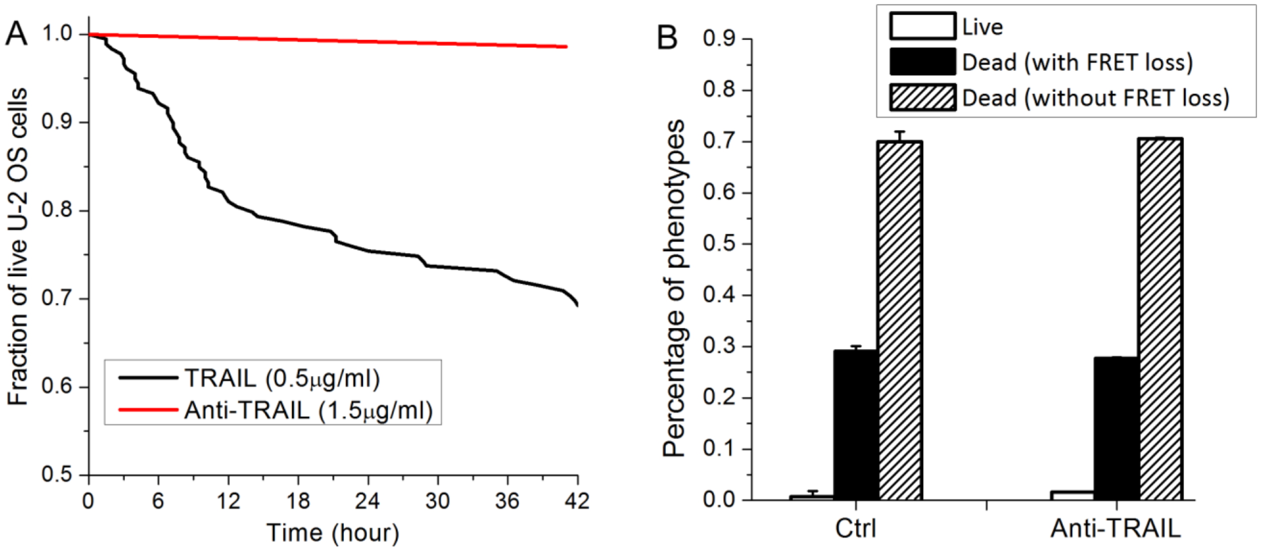

Supplementary Figure S3: Blocking TRAIL-mediated extrinsic apoptosis did not attenuate granzyme-B independent target cell killing by primary NK cells. (**A**) Cumulative survival curves of U-2 OS cells under treatment of 0.5 μg/ml TRAIL or TRAIL plus 1.5 μg/ml anti-TRAIL neutralizing antibody. The data demonstrated that 1.5 μg/ml anti-TRAIL antibody is sufficient to inhibit TRAIL-mediated cell death. (**B**) Comparison of granzyme-B and non-granzyme-B dependent target cell death quantified from U-2 OS cells in co-culture with primary NK cells (after 3-day cultured in IL-2) with or without 1.5 μg/ml anti-TRAIL neutralizing antibody. Data were averaged from 2 independent imaging experiments and the number of cells analyzed ranges from 49 to 88. Error bars: Standard deviations. The data showed that blocking TRAIL-mediated extrinsic apoptosis did not attenuate granzyme-B independent killing by primary NK cells, indicating the death ligand, TRAIL, did not contribute significantly to the primary NK cell cytotoxicity that we observed.

Supplementary Movie SM1: Phase-contrast time-lapse movie of fresh primary NK cells in co-culture with U-2 OS cells (supplemented with high IL-2, 50 ng/ml). Time is indicated in unit of day:hour:minute. Cells were imaged every 10 minutes. See Supplementary_Video1

Supplementary Movie SM2: Fluorescent time-lapse movie of 3-day IL-2 cultured primary NK cells in co-culture with U-2 OS cells (high IL-2 supplement). Images were overlay of the CFP and YFP fluorescence from the granzyme-B FRET reporter as well as the red fluorescence from lytic granule marker, lysobrite. Time is indicated in unit of hour:minute. Cells were imaged every 2 minutes. See Supplementary_Video2

Supplementary Movie SM3: Fluorescent time-lapse movie of 3-day IL-2 cultured primary NK cells in co-culture with U-2 OS cells (high IL-2 supplement) under treatment of 0.8 mM EGTA. Images were overlay of the CFP and YFP fluorescence from the granzyme-B FRET reporter as well as the red fluorescence from lytic granule marker, lysobrite. Time is indicated in unit of hour:minute. Cells were imaged every 2 minutes. See Supplementary_Video3

Supplementary Movie SM4: Fluorescent time-lapse movie of 3-day IL-2 cultured primary NK cells in co-culture with U-2 OS cells (high IL-2 supplement). Images were overlay of the CFP and YFP fluorescence from the caspase-8 FRET reporter as well as the red fluorescence from lytic granule marker, lysobrite. Time is indicated in unit of hour:minute. Cells were imaged every 2 minutes. See Supplementary_Video4
